# Use of vein‐viewing device to assist intravenous cannulation decreases the time and number of attempts for successful cannulation in pediatric patients

**DOI:** 10.1002/pne2.12009

**Published:** 2019-10-31

**Authors:** Alka Sara Saju, Lilly Prasad, Menaka Reghuraman, Immanuel Karl Sampath

**Affiliations:** ^1^ Department of Paediatric Nursing Christian Medical College Vellore Tamil Nadu India; ^2^ Department of Paediatric Surgery Christian Medical College Vellore Tamil Nadu India; ^3^ Present address: Maidstone and Tunbridge Wells NHS Trust Pembury UK

**Keywords:** behavioral distress, fear, pain, pediatric, peripheral cannulation, vein‐viewing device

## Abstract

Every child who contacts a healthcare setting has a potential for intravenous cannulation (IV) procedure and related pain, fear, and distress. Many of the healthcare professionals recognize that there is a lack of intervention to prevent multiple cannulation attempts and to reduce pain and distress inflicted to children during IV cannulation. A quasi‐experimental study was undertaken in pediatric patients to study the effect of a vein‐viewing device (VTorch) on IV cannulation procedure. The number of cannulation attempts and time taken for successful cannulation were assessed with the use of this device (experimental group, n = 159) and compared it with the standard procedure (control group, n = 159). The effect of this device in cannulation associated pain, fear, and behavioral distress were also evaluated among these children. Using Vein‐viewing device as an aid for IV cannulation significantly reduced the time taken for cannulation (*P* = .003) and the number of cannulation attempts (*P* = .03). In addition, there was a significant increase in the first‐attempt cannulation success rate with the use of this device (*P* = .04). The use of vein‐viewing device did not have any direct effect on cannulation associated pain, fear, or behavioral distress among the study participants. The results of this study may aid in improving the quality of intravenous access procedure in pediatric patients.

## 1 INTRODUCTION

Intravenous (IV) cannulation is one of the most common procedures done on hospitalized children. It is a prerequisite for emergency situations, for rehydration, administering systemic drugs, prior to surgeries, and anesthesia. In many situations, a fast and efficient venous cannulation is necessary for patient management. However, IV cannulation is often a difficult experience for children, caregivers, and parents.^1^ Various studies on routine venous access procedures have demonstrated high levels of distress experience among adolescents, schoolers, preschoolers, and toddlers.^2^, ^3^ The major cause of distress among pediatric patients is the pain associated with cannulation. The use of nonpharmacological interventions like ice cap, diversion therapy, hypnosis, parental holding, play therapy, and pharmacological interventions like use of nitrous oxide inhalation, local infiltration with anesthetics and use of topical anesthetic agents are commonly used to alleviate pain during this procedure.^4^, ^5^ However, in most cases due to fear, restlessness among children and small veins, it often requires multiple cannulation attempts to obtain a patent intravenous access. Multiple attempts for cannulation can further increase the experience of pain and discomfort in children. Children may be subjected to physical restraint or the use of force to achieve this.^6^ In addition, multiple cannulation attempts can lead to arterial puncture, thrombophlebitis, skin, and soft tissue injury.^7^ Nurses spend a considerable amount of their time in IV cannulation procedure in children, and it often requires the involvement of phlebotomists or experienced nurses or doctors to perform this procedure. Therefore, a delay in carrying out the treatment can occur. The average time requirement for peripheral IV cannulation is reported as 2.5‐16 minutes, with difficult IV access requiring approximately 30 minutes.^8^ Various interventions to improve peripheral venous insertion success rates use traditional methods to improve visibility & palpability of peripheral veins. This includes tapping the skin, use of betadine solution, warming catheter insertion site, and application of tourniquet.^9^

Currently, new devices with the advanced visualization technologies like ultrasound, transillumination, light‐emitting diode (LED) light waves, and near‐infrared lighting are employed to enhance the visibility of veins and minimize cannulation attempts during an IV cannulation.^10^, ^11^, ^12^ Use of these devices has shown to significantly reduce the IV procedure time and the parents of children rated nurses as having significantly more skill as compared to the group that did not use vein viewer.^13^, ^14^, ^15^, ^16^

VTorch is a commercially available vein‐viewing device which works based on LED technology.^17^ This handheld vein‐viewing device produces a ring of bright light which is focused down and to the center of the ring. When the device is placed on the skin, the light uniformly illuminates the superficial tissues inside the ring. De‐oxygenated blood in veins absorbs the light and appears as dark lines which help in easier visualization of veins. At present, there is no published evidence for the effectiveness of this device in assisting IV cannulation in a pediatric population. We hypothesized that the use of this device would increase the cannulation success rate in pediatric patients. The primary outcome of the study was to investigate the use of VTorch in improving the number of cannulation attempts and time taken for cannulation. We also looked for the effect of this device on pain, fear, and behavioral distress experienced among children undergoing IV cannulation.

## 2 METHODS

This was a prospective, unblinded, quasi‐experimental study. The study was conducted over a period of 6 weeks in the pediatric medical and surgical outpatient departments and pediatric general wards at a tertiary hospital. Sample size was calculated to show a significant difference, with 80% power and 5% level of significance, for three different outcomes—mean number of cannulation attempts, time taken for cannulation, and pain score.^10^, ^18^, ^19^ The calculated sample sizes were found to be 63, 36, and 159, respectively. Among the three, the highest number of 159 for each group was chosen as the sample size for the study. Pediatric patients who were aged between 6‐12 years, who required IV cannulation and fulfilled the inclusion criteria were enrolled in to the study. Patients who required emergency medical management, known case of bleeding disorders, mentally challenged and those on mechanical ventilation and sedation were excluded from the study. The study was approved by the hospital's Institutional Review Board (IRB No: 10796 dated 23.8.2017).

The IV cannulation procedure was performed by staff nurses who had a minimum of 5 years of experience in pediatric wards. Before the commencement of the study, the investigator educated the staff nurses with the use of vein‐viewing device and provided hands‐on training. Study subjects were selected using convenient sampling technique. Children who required IV cannulation were identified by the nurses. The Investigator obtained assent from the children and informed consent from the parents as required. In order to avoid selection bias, the first 159 subjects enrolled for the study were allocated to control group and the next 159 to the experimental group. Children who underwent IV cannulation, where cannulations were done under normal lighting as performed regularly in wards (standard procedure) were taken as the control subjects. The experimental group included those patients who underwent IV cannulation where VTorch vein‐viewing device was used as an aid to visualize veins. Parents were allowed to remain with the children and reassure them throughout the procedure. Pharmacological interventions of pain management were not used during the cannulation procedure. The number of cannulation attempts were obtained by counting the number of times the cannula entered the skin so as to achieve a patent IV access. Time taken for cannulation was assessed using a timer, from the time the tourniquet was tied, till the cannula was successfully flushed with normal saline.

We also assessed the pain, fear, and behavioral distress experienced by the children during the cannulation procedure. Pain was assessed using Wong Baker's faces pain assessment scale.^20^ This scale has pictures of “faces” indicating the levels of pain, with scores from 0 to 10 where 0 indicates “no hurt” and 10 indicates “hurts worst.” Fear was assessed using Children's fear scale, which contains pictures of “faces” indicating increasing degrees of fear, which ranged from 0 to 4.^21^ Once the cannula was placed successfully, children were asked to point to the face that exactly describes the pain and fear they experienced during the procedure. This was done within 2 minutes after completion of the procedure. The behavioral distress was assessed by the investigator using the revised Procedure Behavior Rating Scale (PBRS‐R) during the three stages of the procedure.^22^ Stage 1 includes the time when the child entered the room. Stage 2 is the period when the cannula punctures through the skin till securing the cannula with adhesives, while stage 3 begins 2 minutes after fixing the cannula. The instrument consists of 11 distress behaviors: cry, cling, pain, scream, stall, flail, refusal position, restrain, muscular rigidity, emotional support, and request for termination. These are scored as present or absent over three periods of the procedure. The total PBRS‐R scores can vary from 0 to 33, with higher scores representing greater distress.

Statistical analysis was carried out using Statistical Package for Social Scientists (SPSS), version 16.0. The Mann‐Whitney *U* test was used to compare between groups. Categorical data between the groups were compared using Chi‐squared test. Correlational analysis was done by Spearman's correlation test. A *P* value < .05 was considered significant in all cases.

## 3 RESULTS

A total of 318 IV cannulation procedures were assessed within a time period of 6 weeks. Mean age of children and distribution of male and female participants were similar in control (n = 159) and experimental groups (n = 159) (Table 1). Numbers of previous IV cannulation exposure in children were comparable between the two groups (Table 1). Collectively, these results suggest that the demographic variables were similar in the control and experimental group, and thus, the study participants were homogenously distributed among the two groups. The working experience of nurses who performed the cannulation and the number of cannulations performed by them in a month were also similar in two groups (Table 1).

**Table 1:** Demographic and clinical variables of study participants

Variables	Control group (n = 159)	Experimental group (n = 159)	*P* value
Age (Mean ± SD)	8.74 ± 2.45	8.40 ± 2.32	.197
Sex			
Male, n (%)	91 (57.2)	92 (57.9)	.910
Female, n (%)	68 (42.8)	67 (42.1)	
Number of previous intravenous cannulation exposure in children (Median [IQR])	2.0 (0.0, 5.0)	2.0 (0.0, 5.0)	.919
Number of cannula used for cannulation procedure (Mean ± SD)	1.38 ± 0.69	1.20 ± 0.45	**.007**
Size of cannula used (Median [IQR])			
22G, n (%)	26 (16.4)	21 (13.2)	.430
24G, n (%)	133 (83.6)	138 (86.8)	
Nurses’ working experience (y) (Median [IQR])	14.0 (9.0, 21.0)	12.0 (8.0, 17.0)	.164
Nurses’ experience on intravenous cannulation (No per month, Median [IQR])	100.0 (50.0, 150.0)	100.0 (50.0, 150.0)	.213

Note

For comparison between groups, Chi‐square test was used for categorical data (sex and size of cannula) and Mann‐Whitney *U* test was used for other variables. Results are shown as mean ± standard deviation (SD) or median (interquartile range [IQR]). A *P* value <.05 was considered significant in all cases.

Majority of cannulations were done using 22G sized cannula in both the experimental and control group. The number of cannula used for intravenous cannulation was significantly lower in experimental group, when compared to control group. Frequently used sites of cannulation were brachial, dorsal metacarpal, cephalic, radial, basilic, and medial veins. Distal veins are usually selected for cannulation so as to preserve the proximal veins for future cannulations. If a proper cannulation is not possible in distal site, other proximal veins will be used. In the present study, it was found that 60% of IV cannulations were successful in dorsal metacarpal veins when vein‐viewing device was used to assist cannulation. The successful IV cannulation in dorsal metacarpal veins was only 36% in the control group.

### 3.1 Effect of vein‐viewing device on the number of cannulation attempts and time taken for IV cannulation

**Figure 1:**
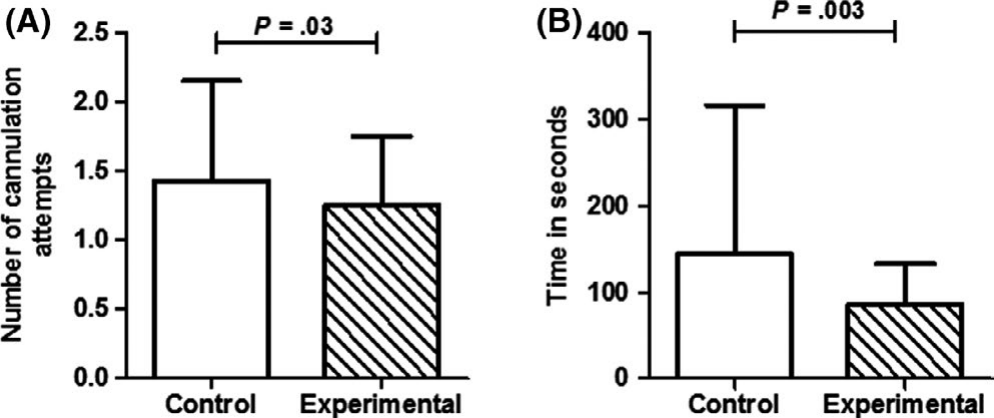
(A) Number of IV cannulation attempts and (B) time taken for successful cannulation in the control group and patients that used the vein‐viewing device (experimental group). Data are shown as mean ± standard deviation (SD)

**Figure 2:**
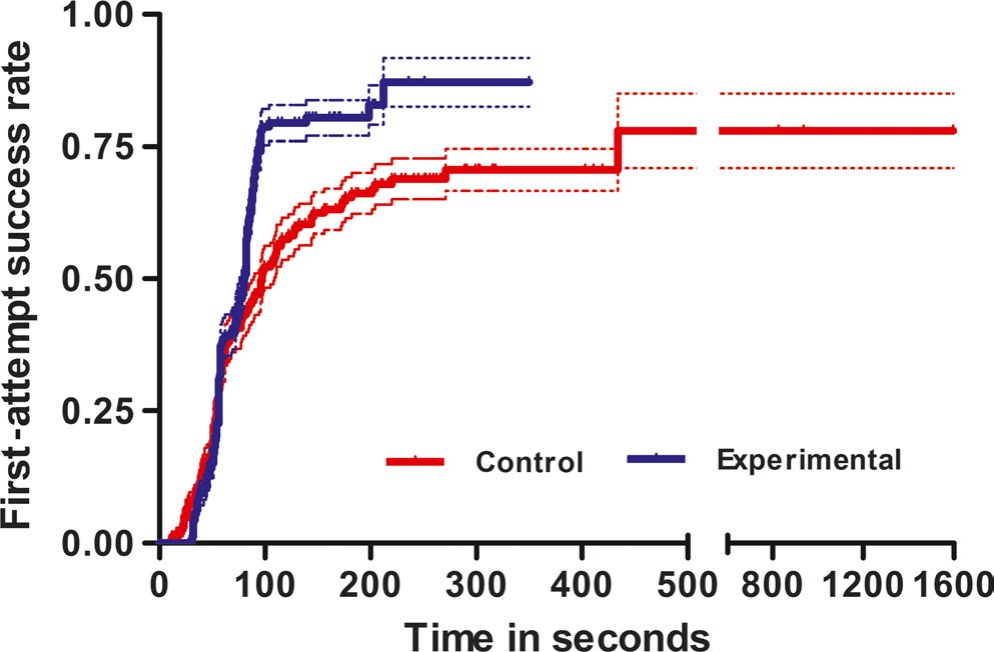
Hazard function plot with 95% confidence intervals (dotted line), showing the first‐attempt success rate in IV cannulation among control (red line) and experimental group (blue line)

Utilization of the vein‐viewing device to assist IV cannulation significantly reduced the number of cannulation attempts in the experimental group, when compared to the control group (Figure 1A). The first‐attempt success rate of intravenous cannulation was significantly higher with the use of vein‐viewing device (77.4%, n = 123), when compared to control group (67.3%, n = 107) (*x^2^* = 4.0, *P* = .04). Success in two attempts was 25.8% in control group and 20.8% in experimental group. The number of cases which required more than two attempts for successful cannulation was 11 (6.9%) in control group and 3 (1.8%) in the experimental group. In patients on whom vein‐viewing device was used, IV placement was 1.6 times more likely to be successful in the first attempt (Odds ratio 1.66; 95% confidence interval [CI]: 1.01‐2.73). The mean time taken for successful cannulation in control group was 144.3 seconds, whereas, in the experimental group, it was 85.1 seconds. Hence, the difference in time for cannulation between two groups was 59.2 seconds. These differences were statistically significant (*P* = .003) (Figure 1B). As shown in Figure 2, a hazard function plot also showed that the first‐attempt cannulation success rate was more and the time taken for cannulation was less in the experimental group, compared to control group.

We further analyzed the relationship of time taken for cannulation with the nurses’ experience in IV cannulation procedure in control and experimental group. In the control group, as the experience of nurses increased, there was a 0.18 seconds decrease in the time taken for successful IV cannulation (95% CI: −0.67, −0.47, *P* = .024). Whereas, in experimental group, the experience of nurses on IV cannulation procedure did not have a major effect on the time taken for IV cannulation (β coefficient 0.04, 95% CI: −0.80, 0.14, *P* = .59).

### 3.2 Effect of vein‐viewing device on pain, fear, and behavioral distress associated with IV cannulation

Cannulation procedures are often associated with pain, fear, and distress among children. We assessed whether the use of vein‐viewing device to assist IV cannulation improved these parameters. Distribution of pain and fear score among study subjects are shown in Tables S1 and S2. The scores of pain during IV cannulation were similar in control and experimental groups (Table 2). The mean value for fear score tended to be lower in experimental group than the control group, but these decreases were not statistically significant (*P* = .08). The score for total behavioral distress experienced during cannulation procedure in control and experimental groups were comparable (*P* = .21) (Table 2). Correlational analysis showed that in the control group, pain and fear scores were positively correlated with the number of IV cannulation attempts (Table 3). A significant positive correlation was also seen between the pain score and time taken for the IV cannulation. Such correlations were not seen in the experimental group (Table 3).

**Table 2:** Distribution of pain, fear, and behavioral distress scores in control and experimental groups

Variable	Control group (n = 159)		Experimental group (n = 159)		*P* value
	Mean ± SD	Median (IQR)	Mean ± SD	Median (IQR)	
Pain during cannulation	5.4 ± 2.9	4 (2, 8)	5.21 ± 2.9	4 (2, 8)	.55
Fear during cannulation	2.3 ± 1.2	2 (1, 3)	2.03 ± 1.2	2 (1, 3)	.08
Total behavioral distress	11.5 ± 7.5	11(4, 18)	10.33 ± 7.4	8 (4, 17)	.21

Note

Pain and fear during cannulation procedure were assessed by Wong Baker's faces pain assessment scale and Children's fear scale, respectively. Procedural Behavioral Rating Scale‐Revised (PBRS‐R) score was used to assess the behavioral distress in patients. Results are shown as mean ± standard deviation (SD) and median (interquartile range [IQR]).

**Table 3:** Correlational analysis of pain, fear, and distress with number of cannulation attempts and time taken for cannulation

Variable	Number of cannulation attempts		Time taken for cannulation	
	Control group (*r*)	Experimental group (*r*)	Control group (*r*)	Experimental group (*r*)
Pain during cannulation	.213**	.147	.209**	.007
Fear during cannulation	.229**	.004	.136	−.045
Total behavioral distress	.049	.088	.099	−.063

Note

Spearman's correlational coefficients are reported. “*r*” shows the correlation coefficient.

** *P* value <.01.

## 4 DISCUSSION

The present study was carried out to assess the use of the vein‐viewing device on assisting intravenous cannulation procedure and its effect on cannulation associated pain, fear, and behavioral distress among pediatric patients. In the experimental group, an LED‐based vein‐viewing device (VTorch) was used as an aid for intravenous cannulation. In the control group, intravenous cannulations were done under normal lighting as per the routine hospital procedure. Intravenous cannulation procedure was assessed for the number of cannulation attempts and time taken for cannulation.

In the present study, it was found that, there was a significant reduction in the number of cannulation attempts, when the vein‐viewing device was used to assist cannulation, compared to the control group. In addition, the successful cannulations within the first attempt were also significantly higher with the use of vein‐viewing device, compared to standard procedure. The present results are consistent with previous studies done using devices with the same working principle. For example, Katsogridakis et al have shown that with the use of Veinlite transillumination device (LED‐based vein‐viewing device) IV cannulation was 2.1 times more likely to be successful in the first attempts, compared to control group (*P* = .03).^12^ In the study done by Hosokawa et al, vein‐viewing device that works with LED lights, facilitated the successful completion of venous cannulations and shortened the time taken for cannulation procedure in pediatric patients.^13^ The above findings together with the findings from our study suggest that LED‐based vein‐viewing devices are useful in minimizing the number of cannulation attempts and therefore can be considered for regular clinical use.

A study done by Carr et al, in 2016,^23^ reported that the experience of healthcare professionals on IV cannulation decreased the time taken for cannulation. This finding was consistent with the finding of the present study, where the time taken for cannulation was lower among experienced nurses in the control group. Another study on pediatric peripheral venous access also reported that the experience of nurses on IV cannulation contributed to improved cannulation success.^24^ This points to the undeniable importance of the skill of healthcare professionals when cannulations are done without the use of any assistive devices. In regular clinical practice, if the less experienced nurses are unable to cannulate, sometimes senior nurses, IV cannulation specialists or doctors will be called for performing the cannulation procedure. But, with the use of the vein‐viewing devices a healthcare professional with a lesser experience in IV cannulation can also be more likely to obtain successful intravenous access without multiple attempts.

The present study also investigated the effect of vein‐viewing device on pain, fear, and distress experienced by children during IV cannulation procedure. We found that the pain scores during the cannulation procedure were similar in control and experimental group. This observation is in agreement with a previous report,^25^ which also suggest that the pain experienced during cannulation procedure was not improved with the use of intravenous assistive devices. Pharmacological methods of pain management, such as use of local anesthesia, during IV cannulation are not routinely carried out in our hospital. Therefore, we have not used such measures in our study. This may account for the moderate pain scores in the present study groups (mean pain scores of 5.4 in the control and 5.2 in the experimental groups). The fear scores during the cannulation procedure tended to be lower with the use of vein‐viewing device compared to the control group; however, these decreases were not statistically significant. Multiple failed attempts to achieve venous access have been shown to increase pain and fear in patients.^26^, ^27^ In agreement with this, in the present study, it was found that the pain and fear scores positively correlated with the number of IV cannulation attempts in the control group. Such correlations were not seen in the group which used the vein‐viewing device to assist cannulation. Use of vein‐viewing device does not have any effect in reducing the behavioral distress experienced during IV cannulation procedure. Collectively, these findings suggest that vein‐viewing device does not have a direct effect in minimizing pain, fear or behavioral distress in children during intravenous cannulation procedure.

There are a few limitations to the present study. The study included only children aged between 6‐12 years, while new‐born, infants, and toddlers were not included in the study. Hence the generalizability of this study in these populations is limited. In the present study, participants were recruited in the control and experimental groups in two time frames, first in the control arm and then in the experimental arm. It may have been a better strategy to use randomized sampling to decrease the possibility of selection bias. Although factors such as age and gender were similar between the two groups, we did not examine the causes of multiple cannulation attempts, such as the diameter of veins, level of dehydration, cannulation difficulty, and body mass index of study participants, and whether these factors were significantly different in the two arms of the study. The other limitation was the fact that it was impossible to blind the person who performed the procedure.

To summarize, the present study indicates that the use of vein‐viewing device to assist IV cannulation significantly reduced the number of cannulation attempts and time taken for cannulation. The first‐attempt success rate for IV cannulation was significantly higher with the use of this device. Use of vein‐viewing device has minimal effect on pain, fear and distress experienced during cannulation. The findings of this study support the use of the vein‐viewing device to improve IV cannulation procedure among pediatric patients and thereby provide better clinical care, avoid complications and reduce procedural delays in starting treatment.

## AUTHOR CONTRIBUTIONS

ASS and L.P designed the study. ASS collected the data, analyzed the results, and wrote the manuscript. MR, IKS critically analyzed results and reviewed the manuscript.
