# Supplementary material for: Use of vein‐viewing device to assist intravenous cannulation decreases the time and number of attempts for successful cannulation in pediatric patients
Source: Paediatr Neonatal Pain. 2019 Oct 31;1(2):39–44. doi: 10.1002/pne2.12009 (PMC8975231; doi:10.1002/pne2.12009)
Supplement: Supplementary file 1 [file PNE2-1-39-s001.docx]

**Supplementary Table 1. Distribution of Pain score assessed using Wong Baker’s Faces pain assessment scale during IV cannulation procedure**

| Group | Control group  n=159 | | Experimental group  n=159 | |
| --- | --- | --- | --- | --- |
|  | n | % | n | % |
| No Hurt (0) | 2 | 1.3 | 3 | 1.9 |
| Hurts little bit (2) | 39 | 24.5 | 45 | 28.3 |
| Hurts little more (4) | 44 | 27.7 | 36 | 22.6 |
| Hurts even more (6) | 23 | 14.5 | 31 | 19.5 |
| Hurts whole lot (8) | 21 | 13.2 | 16 | 10.1 |
| Hurts worst (10) | 30 | 18.9 | 28 | 17.6 |
