# Supplementary material for: Use of vein‐viewing device to assist intravenous cannulation decreases the time and number of attempts for successful cannulation in pediatric patients
Source: Paediatr Neonatal Pain. 2019 Oct 31;1(2):39–44. doi: 10.1002/pne2.12009 (PMC8975231; doi:10.1002/pne2.12009)
Supplement: Supplementary file 2 [file PNE2-1-39-s002.docx]

**Supplementary Table 2 - Distribution of Fear experienced during IV cannulation procedure assessed using Children’s fear scale**

| Group | Control group  n=159 | | Experimental group  n=159 | |
| --- | --- | --- | --- | --- |
|  | n | % | n | % |
| Not scared at all (0) | 5 | 3.1 | 15 | 9.4 |
| Little bit more scared (1) | 45 | 28.3 | 45 | 28.3 |
| A little more scared (2) | 43 | 27.0 | 40 | 25.2 |
| A bit more scared (3) | 32 | 20.1 | 38 | 23.9 |
| Most scared possible (4) | 34 | 21.4 | 21 | 13.2 |
